# Large Area Synthesis of Vertical Aligned Metal Oxide Nanosheets by Thermal Oxidation of Stainless Steel Mesh and Foil

**DOI:** 10.3390/ma11060884

**Published:** 2018-05-25

**Authors:** Fan Wu, Chen Wang, Marvin H. Wu, Kizhanipuram Vinodgopal, Gui-Ping Dai

**Affiliations:** 1 School of Resources Environmental & Chemical Engineering, Nanchang University, Nanchang 330031, China; a1045344165@163.com (F.W.); ncumike@163.com (C.W.); 2 Department of Physics, North Carolina Central University, Durham, NC 27707, USA; mwu@nccu.edu; 3 Department of Chemistry and Biochemistry, North Carolina Central University, Durham, NC 27707, USA; 4 Institute for Advanced Study, Nanchang University, Nanchang 330031, China; 5 Key Laboratory of Poyang Lake Environment and Resource Utilization, Nanchang University, Ministry of Education, Nanchang 330031, China

**Keywords:** metal oxide nanosheet, vertically aligned, three-dimensional networks

## Abstract

We report here the synthesis of metal oxide nanosheets (MONs) directly grown on stainless steel substrates by thermal oxidation in the presence of trace amounts of water. The morphology and microstructure of MONs were characterized by scanning electron microscopy (SEM), transmission electron microscopy (TEM), selected area electron diffraction (SAED), and atomic force microscopy (AFM). The composition of MONs was determined by the energy dispersive system and X-ray diffraction patterns. The results showed that the as-synthesized MONs were ultrathin, vertically aligned, and mostly transparent. They were polycrystalline and were composed primarily of Cr_2_O_3_ and (Fe, Mn)_3_O_4_. The optimal condition to synthesize the MONs with an optimal ultra-high surface atom ratio were determined by varying the temperature and time required for the growth of the MONs. It was found that the lateral size of MONs gradually increases as the temperature rises from 1000 to 1100 °C. An optimal temperature of 1100 °C is obtained in terms of the growth density, size and transparency degree growth morphology, and quality. The structure of MONs changes from two-dimensional to three-dimensional networks when the synthesis time is prolonged to more than 1 h.

## 1. Introduction

Over the past decade metal oxides have sparked tremendous interest, owing to their unique physical and chemical properties, including wide band gap [1,2], reactive electronic transitions [3,4], and good electrical, optical characteristics [5,6,7,8]; this interest continues to grow. Metal oxides are considered to be one of the most fascinating functional materials and have been exploited in versatile practical applications, such as electronics [9], catalysis [10], gas sensors [11], energy conversion, and storage [12]. Also, it has been suggested that properties and functionalities of metal oxides are closely correlated with their sizes, morphologies, and crystalline structures [13,14,15,16]. Thus, there has been an increased awareness of the importance of exploring new features of typical or novel metal oxide materials at the nanoscale level.

Over the past few decades, significant progress has been achieved in the synthesis of metal oxide nanostructures. One-dimensional (1D) metal oxide nanomaterials in the form of rods [17,18], wires [19,20], needles [21,22], and tubes [23] are typical examples of the metal oxide nanomaterials family and have triggered breakthrough achievements in all areas of electronics. More recently, two-dimensional (2D) metal oxide nanosheets (MONs) have emerged as the new members of the metal oxide nanomaterials family and the high surface area of these 2D structures has been exploited in electrochemical applications such as lithium batteries and supercapacitors by numerous groups [24,25]. In recent years, MONs have received a great deal of attention because of their extraordinary physical and chemical properties that impart them with the potential for use in a wide range of applications such as catalytic reactions, sensing, surface-enhanced Raman scattering, and electrochemical capacitors [13,15,26,27]. Therefore, the subject of exploring various novel 2D MONs is of considerable interest. Unfortunately, due to the large specific surface area and high surface energy of metal oxide nanoparticles, they often exhibit a strong tendency to form nanopowders in zero dimensions [9]. Hence, obtaining 2D metal oxide nanosheets with a single- or few-layer atomic thickness remains a challenging task and is highly desirable. Continuous endeavors have been directed towards the synthesis of MONs in the last few years [28,29,30,31,32,33]. Meanwhile, solution-based chemical approaches are regarded as one of the most appealing approaches for the synthesis of 2D MONs. These bottom-up synthetic methods involving templates or surfactants have achieved conspicuous successes in controlling the sizes and morphologies of ultrathin 2D metallic nanosheets. High-quality non-layered α-Ni(OH)_2_ 2D nanosheets are obtained via microwave-assisted liquid-phase growth under low-temperature atmospheric conditions. Subsequently, after heat treatment NiO nanosheets could be synthesized [31]. Nevertheless, solution methods usually involve multiple washing and purification steps [32]. The recent success in the chemical vapor deposition (CVD) synthesis of highly dense hematite nanosheets with extremely large Brunauer–Emmett–Teller (BET) surface areas shows good promise for commercial application, although the synthetic processes require a magnetic field to facilitate the lateral growth [33]. A common thermal oxidation process for the development of the (Fe, Cr)_2_O_3_ oxide layer on the stainless steel mesh with excellent capacitance properties has been recently published [30], in which the developed oxide layer is relatively thicker with a thickness of more than 100 nm and also not transparent or semi-transparent. Notably, cost-efficient and facile synthesis of ultrathin 2D metal oxide nanosheets, especially at a large scale, has been met with limited success and is highly desirable. Therefore, finding innovative and reproducible ways for fabricating these metal oxide nanosheets remains an important challenge.

We report here for the first time a one-step atmospheric pressure water-assisted thermal oxidation process to fabricate ultrathin nanosheets, mainly composed of Cr_2_O_3_ and (Fe, Mn)_3_O_4_, on stainless steel mesh or foil. In this contribution, the metal oxide nanosheets were directly prepared by using a simple and facile technique, consisting of a quartz tube furnace connected to a gas manifold for controlling the gas flow rate and composition. Our experiments show that changes in reaction time and temperature has a great influence on the growth of MONs.

## 2. Experimental Details

### 2.1. Growth of Vertical-Oriented Metal Oxide Nanosheets on Stainless Steel

AISI 304 stainless steel mesh and plate were used for this study, whose chemical composition is listed in Table 1. The materials were ultrasonically cleaned in acetone and ethanol, and blown dry with nitrogen. Prior to growth, the inside walls of the quartz tube were evenly wiped with cotton soaked in deionized water. Then, the samples were introduced at the end of the quartz tube, which was placed in the tube furnace. The temperature of the furnace was raised to the reaction temperature of 1000–1100 °C at a ramp rate of 25–27.5 °C/min under an ambient flow rate of Ar at 30 standard cubic centimeters per minute (sccm). Once the reaction temperature was attained, the quartz tube was moved, thus bringing in a certain amount of water vapor (lying in the direction of the gas inlet in the area out of the heated zone in the quartz tube) as the oxidizer gas. After heat treatment for 5 min to 1 h at 1000–1100 °C in an Ar/H_2_ flow (75% H_2_ by volume) of 40 sccm, the furnace was cooled down to room temperature in a H_2_ atmosphere. The whole process was carried out at atmospheric pressure.

### 2.2. Materials Characterization

Scanning electron microscopy (SEM) experiments were performed using a Hitachi S-4800 instrument (Hitachi, Tokyo, Japan) operating at 20 KV. Selected area electron diffraction (SAED) and high resolution transmission electron microscopy (HRTEM) images were recorded using a JEM-2100 electron microscope (JEOL, Peabody, MA, USA) with an acceleration voltage of 200 KV. Elemental analysis of the specimens was performed with an energy dispersive system (EDS) attached to the transmission electron microscope. X-ray diffraction (XRD, Bruker D8 Advance, Billerica, MA, USA) using Cu/Kα radiation was employed to identify the crystallographic information of the as-obtained sample. Atomic force microscopy (AFM) images were recorded on a 5500 ALP (prototype, Agilent Technologies, Santa Clara, CA, USA) in the tapping mode (dynamic force mode).

## 3. Results and Discussion

**Figure 1:**
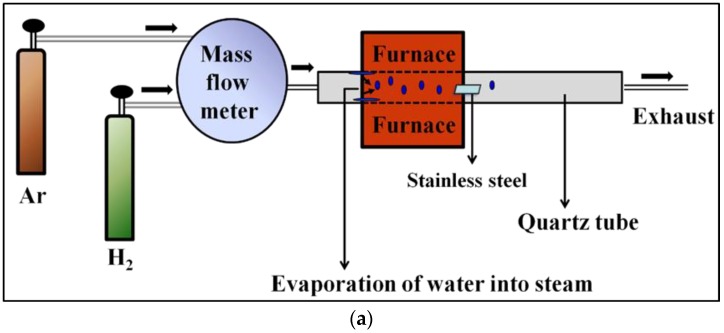

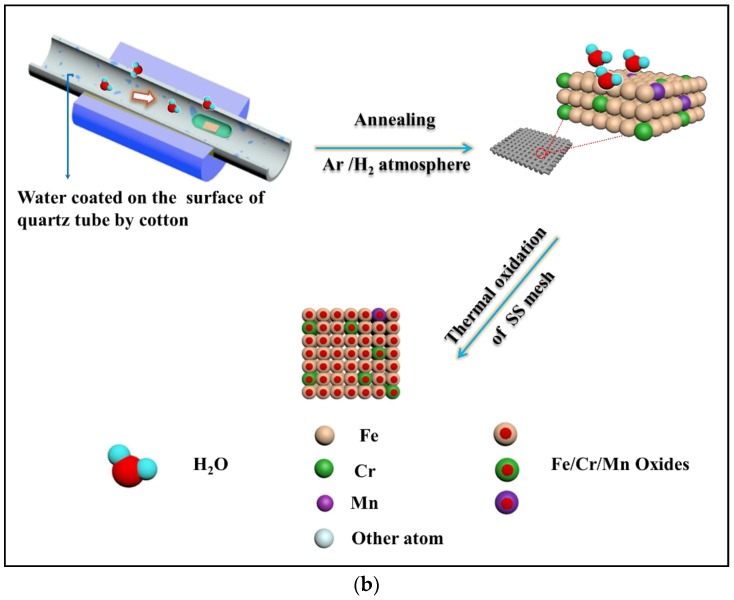
(**a**) Scheme illustration for the metal oxide nanosheets (MONs) grown in the tube furnace; (**b**) is the supplemental presentation of MONs growth process.

Our strategy to fabricate the metal oxide nanosheets (abbreviated as MONs) is illustrated in Figure 1. One step water-assisted thermal oxidation of stainless steel facilitates the formation of Cr_2_O_3_ and (Fe, Mn)_3_O_4_ nanosheets mixed crystalline phase. Here we focus on a systematic investigation of the effect of the experimental conditions and environment on the growth morphology and quality of the specimens.

### 3.1. Effect of Temperature on the Synthesis of MONs

**Figure 2:**
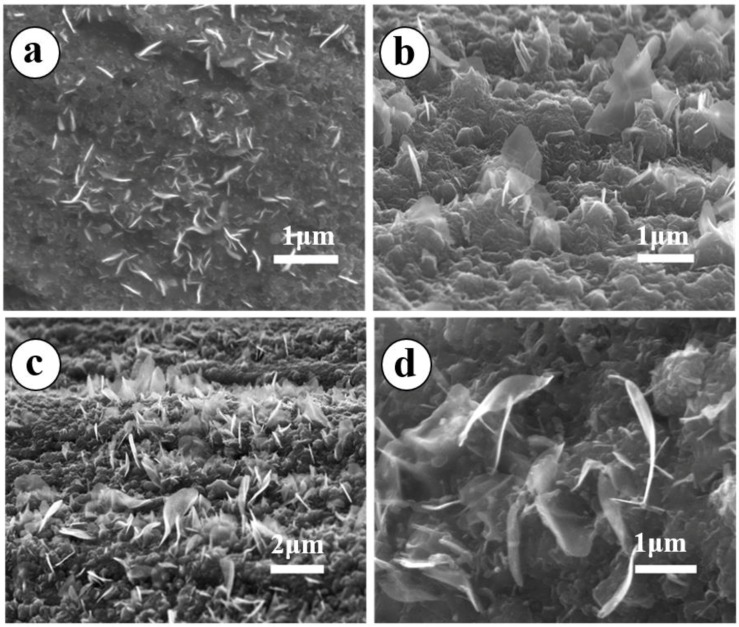
Scanning electron microscopy (SEM) images of MONs grown on the stainless steel at (**a**) 1000 °C; (**b**) 1050 °C; and (**c**) 1100 °C for 5 min each; (**d**) An enlarged view of (**c**).

For the sake of specificity, the first condition to be changed was temperature while the other external conditions were maintained constant. The SEM images were examined to determine the morphology of the as-prepared materials on stainless steel mesh, as shown in Figure 2. There is a noticeable change in the surface morphology when the growth temperature increases from 1000 to 1100 °C. MONs are seen in the samples grown at 1000 °C (Figure 2a) and have an average lateral size of less than 0.5 μm. When the growth temperature is increased to 1050 °C, one observes finer and more aligned MONs (Figure 2b) but with a lower density than in Figure 2a. The lateral size of as-grown MONs in Figure 2b is around 1.2 μm. However, Figure 2c reveals that increasing the annealing temperature further to 1100 °C leads to the formation of highly regular, free-standing, and denser MONs as compared to both Figure 2a,b. An enlarged view of the as-grown MONs is shown in Figure 2d and it can be seen that these nanosheets are ultrathin and transparent. These results as a whole suggest that the growth rate and surface coverage density of MONs are sensitive to temperature, and the optimal growth condition is 1100 °C, which is the maximum temperature in our furnace.

### 3.2. Dependence of MONs Morphology on the Reaction Time

**Figure 3:**
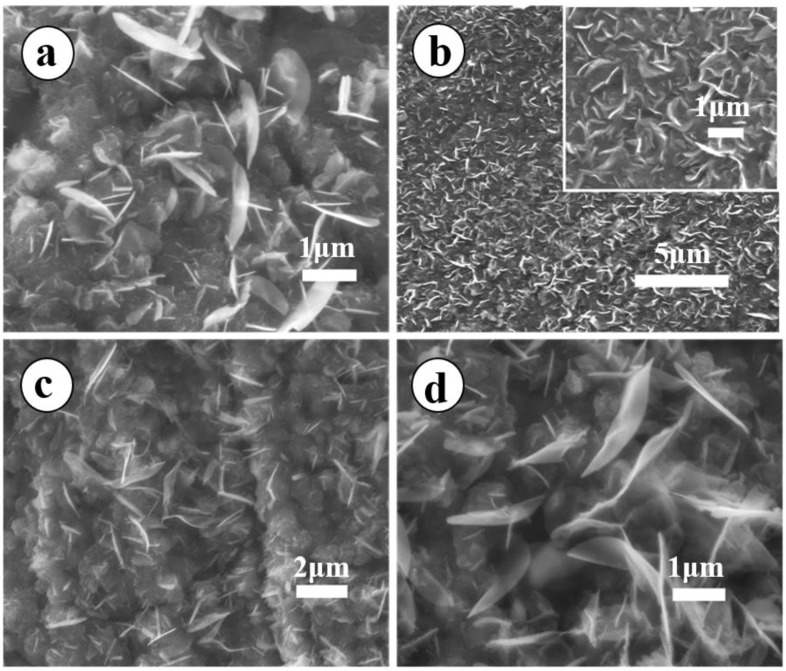
SEM images of MONs grown on the stainless steel at (**a**) 30 min; (**b**) 1 h; (**c**) 2 h; (**d**) 3 h.

In addition to the aforementioned discussion of the influence of temperature on the growth of MONs, reaction time was also regarded as an important parameter in the MONs growth process. Figure 3 shows SEM images obtained at different reaction times while keeping all other parameters constant. As is clearly shown in Figure 3a, very-fine and free-standing 2D MONs with an average lateral size of nearly 1 μm are grown on the substrates after 30 min of growth. However, when the growth time is increased to 1 h (Figure 3b) the surface of the substrate is covered with a denser three-dimensional (3D) MONs networks, with no significant variations in lateral size. Attempts were made to further clarify the effect of annealing time on the growth of MONs by prolonging the growth period for 2 h or 3 h and as is shown in Figure 3c,d, respectively. Extension of these reaction times to 2 or 3 h is accomplished by allowing the system to cool down at the end of 1 h and repeating the entire process, including reintroducing water into the quartz tube. It is noteworthy that both the average lateral size of the MONs in Figure 3c,d are enhanced and are approximately 2.0 μm and 2.8 μm, respectively. Prolonging the growth time increases the thickness and surface area density and lateral size of the as-prepared MONs. It is crucial to stress that a transition from 2D free-standing graphene-like MONs to a 3D network can be clearly seen when the growth time is increased beyond 1 h. 

### 3.3. Microstructure Analysis of the Samples

**Figure 4:**
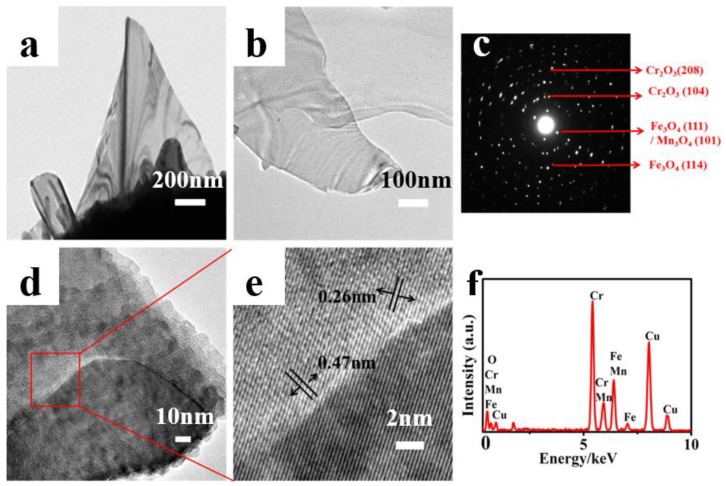
(**a**,**b**) are the typical TEM images of MONs; (**c**) is the corresponding selected area electron diffraction (SAED) pattern; (**d**) High resolution transmission electron microscopy (HRTEM) image of MONs; and (**e**) is the corresponding enlarged HRTEM of (**d**); (**f**) is the energy dispersive X-Ray (EDX) analysis of MONs. All of the images were obtained from samples prepared at a temperature of 1100 **°**C for 1 h.

**Figure 5:**
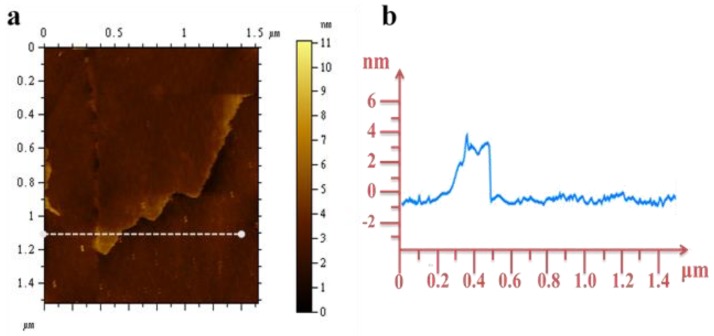
(**a**) Atomic force microscopy (AFM) image of MONs; (**b**) Height profile of the layer across the dotted line in panel (**a**).

To better understand the structures of these nanomaterials, TEM images were collected for detailed structural investigation. The low magnification TEM images in Figure 4a,b display the typical ultrathin graphene-like nanosheets with a thickness of approximately 5 nm. The corresponding SAED pattern shown in Figure 4c indicates the polycrystalline nature of the MONs. The rings corresponding to the lattice plane (111) and (114) can be indexed with Fe_3_O_4_. Similarly, the rings (104) and (208) can be indexed with Cr_2_O_3._ The high-resolution HRTEM image shown in Figure 4d reveals that the as-fabricated MONs consists of two different metal oxides, as can be seen at the enlarged view of Figure 4d in Figure 4e. The oxide stacking can be easily observed, and the internal layer spacing measured as ~0.47 nm and 0.26 nm correspond to the (111) Fe_3_O_4_/(101)Mn_3_O_4_ and (104) Cr_2_O_3_ phase, as shown by the arrow heads. A possible explanation can be attributed to the substrate alloys and composites (i.e., Fe/Cr/Mn/Ni) and/or their oxides [6,15]. The relevant elemental analysis in Figure 4f demonstrates that the as-grown MONs mainly contain element chromium (Cr), iron (Fe), manganese (Mn), copper (Cu), and oxygen (O). (The Cu in this case arises from the copper TEM grid.) In a previous work, hematite and spinel nanostructures, like nanowires or nanoflakes, were successfully synthesized at high-temperatures with the assistance of water [32]. Hence, we similarly conclude that the as-grown MONs in our case consists mainly of Cr_2_O_3_ and spinel (Fe,Mn)_3_O_4_. Figure 5 shows an AFM image, where a planar slice can be clearly observed. The height profile of the nanosheets along the dotted lines in the AFM image yield an average thickness of ~3 nm, confirming that the as-prepared MONs have an ultra-thin layered structure.

**Figure 6:**
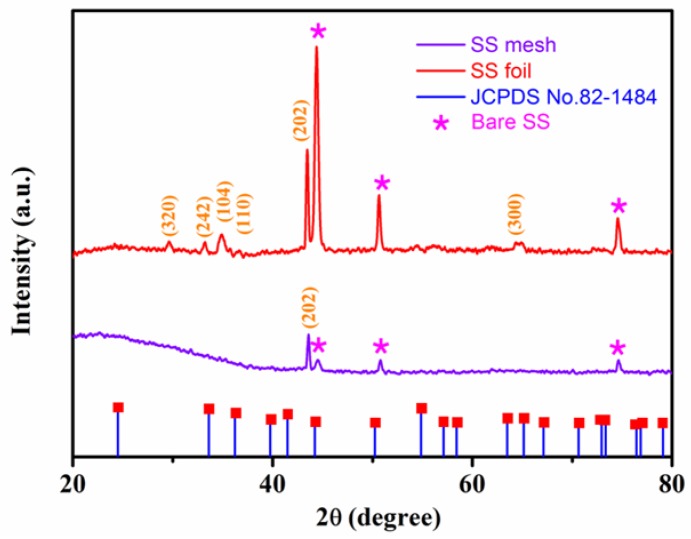
X-ray diffraction (XRD) patterns (reference: JCPDS card No. 82-1484) of the MONs grown on stainless steel mesh and foil in 1100 **°**C for 1 h.

XRD was used to confirm the crystalline phase of the synthetic composite (Figure 6). The peak at approximately 2θ = 44.4° in the as-prepared MONs grown on stainless steel mesh is ascribed to the (202) diffraction mode of Cr_2_O_3_ in agreement with the Joint Committee on Powder Diffraction Standards (JCPDS) card No. 82-1484. As shown in Figure 6, the other three peaks of stainless steel substrate (denoted as SS in pattern) are also visible due to the small thickness of the film. However, we see no evidence of any spinel peaks in the XRD of the mesh. To resolve this, we have grown the MONs on the stainless steel foil for comparison. In consequence, seven new typical characteristic peaks at approximately 2θ = 29.6°, 33.2°, 35.2°, 35.7°, 44.4°, and 64.8° appeared as compared with the XRD pattern of pure stainless steel and are ascribed to the diffraction mode of Mn_3_O_4_ (320), Fe_3_O_4_ (242), α-Fe_2_O_3_ (104), α-Fe_2_O_3_ (110), Cr_2_O_3_ (202), and Cr_2_O_3_ (300), respectively [24,32,33,34,35]. The dominant peak is assigned to Cr_2_O_3_ (202). Based on this comparison, we conclude that the as-synthesized MONs on the stainless steel mesh mainly consist of Cr_2_O_3_ and spinel (Fe, Mn)_3_O_4_, and little Fe_2_O_3_.

**Figure 7:**
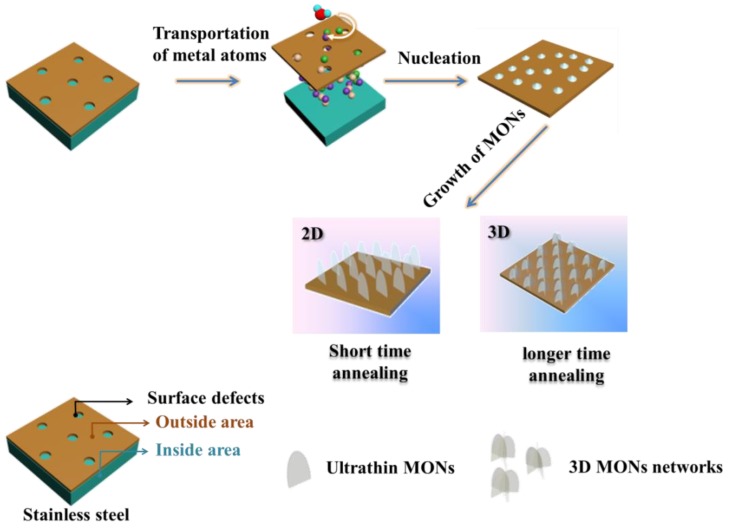
Growth mechanism of MONs in the water assisted high temperature annealing process.

The growth mechanism of MONs synthesized in our thermal oxidation process is consistent with the tip-growth mechanism, proposed by Takagi [36], and the growth mechanism graph was demonstrated in Figure 7. The surface defects or cracks in the stainless steel substrate serves as the nucleation sites for MONs growth, and metal oxide layers with thickness of several micrometers are subsequently formed before the growth of MONs. Iron oxide layers, including those of FeO, Fe_x_O (x > 1), Fe_3_O_4_, and Fe_2_O_3_, and together with chromium oxides or manganese oxides are formed below the surface of the alloy substrate and can serve as the reactants for growth at high temperature. Meanwhile, high-temperature annealing can generate thin oxide layers with larger porosity and more grain boundaries, so that the chromium, manganese, and iron atoms can constantly diffuse to the surface and form nucleation sites for the growth of MONs. The highly energetic metal atoms can easily react with water vapor. Further, the metal oxides formed in the high temperature furnace are directly deposited on the surface of the alloy substrate with robust adhesion, allowing for the subsequent formation of MONs. According to a previous report, a double oxide layer is identified in most of the studies. Steam oxidation leads to the formation of a duplex oxide layer with usually an outside hematite layer and an inside layer composed of spinel oxide. This is in agreement with our aforementioned hypothesis that the outside hematite layer in our sample may be the Cr_2_O_3_, and the spinel oxide may be the (Fe, Mn)_3_O_4_. Deshmukh et al. [34] also mention that in the thermal oxidization process, when the temperature is gradually elevated from 200 to 800 °C, the color of the meshes change. They proposed that this was due to the diffusion of Cr begun in the oxide layer at 500 °C, and Cr rich oxide layer (Brownish black) observed at 800 °C. This is in agreement with our experimental results, where MONs consist of more Cr_2_O_3_ than Fe_3_O_4_ with an annealing temperature of 1100 °C.

## 4. Conclusions

In summary, vertically oriented MONs are directly synthesized by a one-step thermal oxidation process at atmospheric pressure. The preparation method is facile and simple, and the growth of MONs can be controlled by varying the reaction temperature and growth time. The highly regular, free-standing, ultrathin, transparent, and denser MONs are observed by adding trace water under an optimal growth temperature of 1100 °C. The transition from 2D free-standing graphene-like MONs to 3D network is demonstrated with a growth time of beyond one hour. The as-prepared MONs are a duplex oxide layer, mainly composed of Cr_2_O_3_, followed by (Fe, Mn)_3_O_4_, and Fe_2_O_3_. The formation of MONs follows a tip-growth mechanism. As a result, this direct one-step growth of MONs on stainless steel does not require any additional binders or conducting additives, providing a great promise for advances in 2D metal oxide nanomaterials for possible applications, such as catalysis, gas sensor, electronics, energy conversion, and storage.

## Figures and Tables

**Table 1:** Chemical composition of alloys 304L used, in wt. %.

C	N	Si	Mn	S	P	Cu	Co	B	Ni	Cr	Fe
≤0.035	≤0.08	≤1.00	≤2.00	≤0.015	≤0.03	≤1.00	≤0.06	≤0.0018	9.00–10.00	18.5–20.00	Bal.
